# A Case of Ovarian Pregnancy—Laparotomy—Cure

**Published:** 1892-12

**Authors:** Emory Lanphear

**Affiliations:** Kansas City, Mo.


### A CASE OF OVARIAN PREGNANCY—LAPAROTOMY—CURE.

BY EMORY LANPHEAR, M. D., PH. D.,
KANSAS CITY, MO.

Many a woman has gone to the grave with a diagnosis of "pelvic hæmatocele," "peritonitis," "pelvic cellulitis," etc., when the real cause of death was extra-uterine pregnancy. Fortunately we are rapidly coming to the conclusion that (1) extra-uterine pregnancy is quite frequent; (2) that it may be recognized in many cases ; (3) that in modern surgical methods we have a comparatively safe cure. As for the question of "pelvic hæmatocele," from an observation of a number of cases, I am almost convinced that in nearly every instance the trouble is ruptured tubal pregnancy.

It is now pretty generally conceded that all fecundation takes place either in the tubes or on the surface of the ovary, and the wonder has been that ectopic gestation is as rare as generally believed. I do not think it is very rare—in fact, it is of quite common occurrence ; but because of unfavorable environments, early death and absorption of the product of conception occur in a large portion of cases.

Lawson Tait has claimed that all extra-uterine pregnancy is tubal ; in opposition to this Franklin Townsend, of Albany, before the American Association of Obstetricians and Gynæcologists, 1888, presented an elaborate essay (which met general approval), in which the assertion is made that "extra-uterine fœtation can and does occur either in the Fallopian tube (a frequent form), in the ovary, upon the ovary, or in the peritoneal cavity." That true ovarian pregnancy does take place is proven by the following case :

CASE.—Mrs. John W., age 42 years, patient of Dr. T. Brown, of Hamilton, Mo.; two children—one 18 years and one 14 years of age, pregnant four years ago, but miscarried at four months ; never well since; menstruating too freely and suffering from retroversion. Last menstruation occurred the last of May, 1892; some symptoms of pregnancy. August 2, was taken with hæmorrhage from uterus, accompanied by excruciating pains in left ovarian region, the bleeding stopped, but pain continued with great collapse, and the abdomen became distended to its limit. Temperature has ranged from 99½ to 102. In the night of August 15 I first saw her and examined her under chloroform. The whole pelvis was filled with a boggy mass, the lower part of the abdomen very full and a lump the size of a large orange easily made out in the left ovarian region. I therefore felt convinced of the accuracy of Dr. Brown's diagnosis of ruptured tubal pregnancy, and advised operation. On the morning of August 16, assisted by Drs. T. Brown and W. T. Lindley, I made abdominal section, and removed about one and a half gallons of fluid and clotted blood; the left ovary was found to be the seat of pregnancy, its tube being whole and unaffected save that the fimbriated extremity was bound down to the broad ligament by inflammatory action; to the ovary the placental attachment was plainly made out, and in its ruptured envelop the dead fœtus. A clamp was applied, and the broad ligament transfixed and tied with catgut, the tube, remnants of ovary and the baby cut away. The abdomen was then thoroughly irrigated with boiled hot water to the amount of about eight gallons, and the abdomen closed with catgut sutures, without drainage, with the usual dressings. Six hours later the temperature had dropped two degrees and the patient was free from pain for the first time in many days; but little shock. August 20, she was reported as free from pain, sleeping well and appetite returning; temperature normal and pulse 80. Recovery was uneventful, she being allowed to sit up in bed on the tenth day and to walk a little by the sixteenth.

Examination of the specimen showed conclusively that this was a true ovarian pregnancy, the tube being as perfect as any I have ever seen, save at its extremity, as already mentioned.

The treatment usually advised for extra-uterine pregnancy is electricity, if a diagnosis has been made prior to rupture of the enveloping structures, the idea being to destroy the vitality of the impregnated ovum and allow its absorption. This I do not believe to be good treatment, for whenever pregnancy is sufficiently advanced to allow even a provisional diagnosis, the mass is already so large that it can only remain, and remaining be a constant menace to the life of the patient; for no woman with such a foreign body in the pelvis can be for one moment free from danger. Fatal inflammation is likely to be set up at any time, even years afterwards, and if a wrong diagnosis has been made, incalculable mischief may be done by the electric current; whereas, as Wathen says, "We find nothing against the knife, except that it is a surgical operation. No case of death has ever been reported as due to the knife. If a mistake in diagnosis has been made, no harm has been done, as the disease, whatever it is, will require the knife anyway." And, I may add, if the condition happens to be such that nothing has to be removed, exploratory laparotomy is an almost absolutely safe operation in clean and skilled hands; I have opened many bellies for exploration, and have never seen a symptom follow to cause regret. So I will say that whenever ectopic pregnancy is suspected, laparotomy is indicated, and urgently.

All authorities unite in advising abdominal section whenever rupture has occurred. That the operation may be done even a week or two after the accident is proven by the case here reported. But as a rule it may be said, the earlier the surgical interference the better.

1334 *East Eighth Street.*

---

### CHAPPED HANDS.

Dr. Steffen uses the following for rough and chapped hands:

℞ Menthol, gr. xxv.

Salol, ʒ ss.

Olive oil, *m* xxv.

Lanoline, ʒ x.

When this ointment is applied twice a day, the pain quickly ceases, the skin softens, and the rhagades heal.—*Medical Record.*

